# Data of CEO power, chair-CEO age dissimilarity and pay gap of Chinese listed firms

**DOI:** 10.1016/j.dib.2020.106158

**Published:** 2020-08-08

**Authors:** Jiajun ZHU, Jing GAO, Hongping TAN

**Affiliations:** a School of Business, Jiangnan University, China; b Desautels Faculty of Management, McGill University, Canada

**Keywords:** Age dissimilarity, Pay gap, CEO power, Co-working time, CEO ability, Monitoring

## Abstract

This data describes the raw and processed information such as salary, power, and age of the CEO and the chairman between 2009 and 2018 in China's listed firms. The data set contains the data of variables based on the characteristic of the firm, personal, team, and supervision. The dissimilarities and similarities of the characteristics between the chairman and the CEO are the core of this data set. The dissimilarities refer to individual and team differences. Individual differences refer to differences in age, gender, tenure, experience, shareholding, and salary of the chairman and CEO, while team differences refer to differences in team size and the standard deviation of the management board members’ age. The similarities refer to joint tenure and family relations between the chairman and CEO. These variables can be used to estimate the impact of chair-CEO age dissimilarity on the relationship between CEO power and chair-CEO pay gap of the Chinese listed firms through binary probit or multinomial regression.

**Specifications Table**

**Table:** 

**Subject**	Business, Management and Accounting (General)
**Specific subject area**	Executive Compensation, Corporate Governance
**Type of data**	Tables Graphs
**How data were acquired**	This article downloads firm financial data from the database CSMAR and CCER, mines non-financial data from the annual reports, company filings (e.g., appointment notices, performance forecast, and governance reports). Other data are obtained from disclosure reports (e.g., board resolutions, equity incentive plans, and announcements of major events) published on the website of China Securities Regulatory Commission (CSRC). The news of firms reported by paper and electronic media are retrieved from the DATAGO Database.
**Data format**	Raw and processed data file.
**Parameters for data collection**	The data are selected from A-share firms traded on Shanghai and Shenzhen stock exchange between 2009 and 2018. Firstly, due to the particularity of financial indicators, financial firms are excluded from the sample. Secondly, firms with consecutive losses of more than 2 years are not included in the sample. Thirdly, samples of firms with severely distorted or missing data were eliminated. At last, 15,138 unbalanced panel data were obtained across 10 years. The top and bottom 1% of all continuous variables are winsorized.
**Description of data collection**	The factor analysis, correlation analysis, and regression analysis are conducted by using STATA 12.0 software. The graphs are drawn with Origin Pro8 software. CEO ability (MA-Score) is calculated with DEA-SOLVER Pro5 programs. Time: 2009–2018
**Data source location**	China Securities Regulatory Commission (CSRC) City/Town/Region: Beijing Country: China Institution: China Stock Market Trading Database (CSMAR) City/Town/Region: Shenzhen Country: China Institution: China Center for Economic Research(CCER) City/Town/Region: Beijing Country: China Institution: DATAGO Database City/Town/Region: Hong Kong Country: China
**Data accessibility**	Repository name: Mendeley Data Data identification number: 10.17632/sxdm9zkbjs.1 Direct URL to data:http://dx.doi.org/10.17632/sxdm9zkbjs.1
**Related research article**	Zhu, J.J., Gao, J., & Tan, H.P., 2020. How the CEO power and age dissimilarity shape the chair-CEO pay gap: Empirical evidence from China, The North American Journal of Economics and Finance. In press, https:// https://doi.org/10.1016/j.najef.2020.101221

**Value of the Data**

• The data provide insights on the relationship between CEO power, chair-CEO age dissimilarity, and pay gap in Chinese firms. At present, there are few studies and applications on the age difference between the chairman and the CEO, making this data a valuable resource.
• These data will be beneficial for scholars interested in researching the cognitive differences between the chairman and CEO in Chinese firms.
• These data can be analyzed and/or compared with data in other emerging economies. They can be used to recognize cognitive conflicts caused by age dissimilarity, or as basic data to help the listed firm identify the role of similarity and heterogeneity of executive and team characteristics, and improve salary or equity incentives in corporate governance.

## 1 Data description

The data of this research describe how the chair-CEO age dissimilarity and CEO power affect the chair-CEO pay gap, spanning the period between 2009 and 2018 in China's listed firms. The sample firms were randomly selected each year and accounted for more than 1/3 of the total number of listed firms that year. These samples include firms listed on the Main-Board Market, Small and Medium Enterprise Board, and Second-board Market. Around 29.2% of the firms are state-owned or state-controlled. The final sample is comprised of a nonbalanced panel of 15,138 firms across 10 years. The raw data are downloaded from the CSMAR and CCER databases. Paper and electronic medias’ news of firms is retrieved from the DATAGO Database. Most of the non-financial information is hand-collected by reading the annual reports, company filings (e.g., security prospectuses and governance reports) filed on the websites of listed firms. Other data come from information disclosure (e.g., board resolutions, equity incentive plans, and announcements of major events) on the website of China Securities Regulatory Commission (CSRC). The calculation is made via Excel to form the executives and firm characteristics. STATA 12.0 is used to conduct factor analysis, correlation analysis, and regression analysis.

This data set uses regression equations to analyze the effects of age dissimilarity and CEO power on the pay gap between chairman and CEO. The dependent variable is the pay gap between chairman and CEO. The compensation of the CEO or the chairman is calculated by the comprehensive annual salary earned in one year, which includes total wages, allowances, funds, bonuses, stock options granted (Black-Scholes value), restricted stocks granted (market value), and all other income. The comprehensive compensation is denominated in RMB, and the foreign currency incomes of executives are converted into RMB with the exchange rate at the end of the year. The chair-CEO pay gap is the natural logarithm of the absolute value of the total pay difference between chairman and CEO plus one in the year. Therefore, the chair-CEO pay gap is calculated and processed. The main independent variable is the age dissimilarity between the chairman and CEO which is measured by three ways proposed by Goergen et al. [1]: Chair-CEO age gap, Chair-CEO age ratio, and Generational gap. The Chair-CEO age gap is the age of the chair minus the age of the CEO. Chair-CEO age ratio is the age of the board of directors divided by the age of the CEO. The generational gap is a dummy variable that equals one if the age gap between the chairman and the CEO is greater than or equal to 10 and zero otherwise.The three indicators are calculated on the raw data of the age of the chairman and CEO. Since China has no strict restrictions on the age of executives of listed firms, the oldest chairman is 85 years old and the youngest is 26 years old, while the oldest CEO is 80 years old and the youngest is 24 years old in the sample data. The average age of CEOs was 49.011 years, while the average age of chairmen was 52.789 years. Based on the calculations, the mean age gap (Chair-CEO age gap) is 3.841 years and the mean age multiple between the chairman and the CEO (Chair-CEO age ratio) is 1.090. The generational gap between the chairman and the CEO (Generational gap) was found in 23.7% of sample firms. The chairman is younger than the CEO in 41.1% of the sample firms. The distribution of the age difference is similar to the distribution of the age difference between the chairman and CEO of German listed firms studied by Goergen et al. [1], which has certain theoretical significance for the correlation and regression analysis of age differences. The age dissimilarity reflects two notable characteristics. First, there is a significant age difference between the chairman and CEO, but due to the limitations of domestic senior executives' tenure and governance structure, the age difference more than 20 is relatively small. Second, there is a certain skew structure in the age difference, that is, the chairman is generally older than the CEO. CEO power is another important independent variable. It is measured by a composite score of factor analysis based on seven variables, including duality, shareholding, tenure, education, relatives, political relationship, and independence of the board. These variables represent structural power, ownership power, expert power, and prestige power from multiple angles [2]. These seven variables used to measure CEO power are all assigned values of 0 and 1, and the overall score is calculated through factor analysis. The overall score is used as the value of CEO power. The bigger the score, the higher the CEO power.

CEO ability and joint tenure are two categorical variables used to compare the effects of ability and relationship on age dissimilarity. CEO ability (MA-Score) is calculated by the data envelopment analysis (DEA) method which was developed by Demerjian et al. [3]. CEO ability is measured in two stages. In the first stage, the DEA statistical procedure is used to generate firm efficiency scores based on seven inputs (cost of goods sold, selling and administrative expenses, net fixed assets, net operating leases, net research and development, purchased goodwill, and other intangible assets) and one output (sales). In the second stage, firm efficiency scores calculated with the DEA method are employed as a dependent variable in a Tobit regression model by industry. In this model, the independent variables include firm size, market share, positive free cash flow, firm age, business segment concentration, foreign currency indicator, and year indicator. The residual from the Tobit model is the CEO ability score, which is estimated to separate CEO ability factors from firm characteristics. We adjusted the CEO ability score based on industry to generate a categorical variable CAD to compare the impact of CEO ability on the effect of age dissimilarity. CAD is a dummy variable set to one if the CEO ability score is greater than the annual median of the industry, and zero otherwise. Joint tenure is defined by the co-working time (years) between the chairman and CEO. It is an important indicator to study the similarity between the chairman and the CEO. The joint tenure is calculated based on the raw data of the tenure of the chairman and CEO. The descriptive statistics of the sample show that the longest tenure of the chairman is 28 years and the shortest is 0.08 years, while the longest tenure of CEO is 17.8 years and the shortest is 0.08 years. And the average tenure of CEOs was 3.53 years, while the average tenure of chairmen was 3.82 years. Thus, the average joint tenure between the chairman and the CEO is 2.516 years, while the longest co-working time is 13.417 years. Similarly, we adjusted the joint tenure according to industry to generate a categorical variable WTD to compare the impact of the relationship between chairman and CEO on the effect of age dissimilarity. WTD is a dummy variable set to one if the co-working time between the chairman and CEO is longer than one term (3–4 years), and zero otherwise.

Following Goergen et al. [1], we control for firm characteristics, CEO characteristics, chair characteristics, team characteristics, and internal and external supervision characteristics. Firm characteristics include total assets (logarithm of the company's total assets), the firm listed age (logarithm of years since listed), book leverage (total liabilities over total assets), type of firm (a dummy variable that equals one if state-owned or state-controlled firms, and zero otherwise), ROE (return on equity), stock return rate (abnormal return after adjustment of A-share market index), risk(beta coefficient of firm's stock), R&D(annual R&D expenditures divided by total revenue). CEO characteristics include CEO age and CEO tenure which is the number of years the CEO has been serving as the firm's CEO. Founder CEO is a dummy variable equaling one if the CEO founded the firm. Similarly, chair characteristics include the chair age, chair tenure, and founder chair. Besides, we also consider another important characteristic variable of the chairman - busy chair, which is a dummy variable set to one if the chairman of the supervisory board holds three or more directorships, and zero otherwise. Other chair–CEO dissimilarities and similarities include gender gap, tenure gap, experience gap, shareholding gap, and same family which is a dummy variable set to one if the chairman and the CEO are from the same family. Team characteristics include pay growth (the average salary growth rate of the executive team), team size gap (the size difference between executive and board team), the standard deviation of management board age, management stability (the change rate of people in the executive team), board stability (the change rate of people in the board), and busy board (dummy variable set to one if at least 50% of the shareholder representatives hold three or more directorships, and zero otherwise). Supervisory characteristics are divided into internal and external supervision. Internal supervision characteristics include board meetings and supervisory board size. External supervision characteristics include analyst's attention, institutional investors' attention, and media's attention.

## 2 Experimental design, materials and methods

### 2.1 CEO power and the pay gap

The impact of CEO power on its salary incentives is a hot topic in management power theory research. Therefore, we consider that CEO power is negatively related to the chair–CEO pay gap, that is, powerful CEO can reduce the pay gap between chairman and CEO [4, 5]. Thus, in this hypothesis, CEO power is an independent variable, and the pay gap between chairman and CEO is a dependent variable. According to the Finkelstein [2] method, seven variables are used to represent the characteristics of CEO power. Based on factor analysis, three main factors from seven variables are extracted. The total contribution rate of three principal components is 70.23%, while the KMO (Kaiser-Meyer-Olkin) value of 0.583 and the p-value of the Bartlett test is 0.000, which meet the basic requirements of factor analysis. The combined scores of the three main factors are used to measure CEO power. Firstly, CEO power is used as an independent variable to confirm the effect of CEO power on the chair-CEO pay gap. CEO power's square (i.e., Squared CEO power) is used to confirm that there is a linear relationship between CEO power and the chair-CEO pay gap. In order to verify the robustness of CEO power, PD1(r (see, e.g., [6]), PD2(see, e.g., [7]) and PD3(see, e.g., [8]) were established to serve as proxies for the CEO's power. In addition, chair-CEO pay gap is replaced with the CEO-TMT pay gap(i.e., the natural logarithm of the absolute value of the pay difference between the CEO's salary and other executives’ average salary) to test the impact of the CEO power on the pay gap between CEO and other executives. It helps to observe the role of CEO power on salary incentives from another perspective, that is, CEO power can reduce the pay gap between the chairman and the CEO and widen the pay gap with other executives.

### 2.2 Chair-CEO age dissimilarity, CEO power, and the pay gap

The impact of age dissimilarity on the pay gap between the chairman and the CEO and the moderating effect on the relationship between the CEO power and pay gap are the focus of our research. Therefore, we consider that the chair-CEO pay gap is positively related to age dissimilarity. It also significantly weakens the impact of the CEO power on the chair-CEO pay gap, that is, cognitive conflicts caused by the age dissimilarity has a certain inhibitory effect on the CEO power. Thus, in this hypothesis, age dissimilarity is used as the independent variable as well as the moderating variable, and the pay gap between chairman and CEO is the dependent variable. Following Goergen et al. [1], Chair-CEO age gap, Chair-CEO age ratio, and Generational gap are used to describe the age dissimilarity between the chairman and the CEO. Chair-CEO age gap and Chair-CEO age ratio measure the age dissimilarity from the perspective of absolute value and relative value respectively. The method to measure the Chair-CEO age gap is the same as Goergen's [1] method, but the definition of the generational difference is somewhat different. The definition of the generational gap as 10 years is mainly due to the following two considerations [9]: On the one hand, due to cultural and geographical differences, China's generational differences are usually measured by 10 years rather than 20 years defined in traditional sociology. Post-60 s, post-70 s, and post-80 s became synonymous with generational differences. If managers are the same age, they will have similar ways of thinking, values, and codes of conduct. On the other hand, restricted by the employment period and promotion, the age difference between the CEO and chairman of Chinese listed firms cannot be too great, especially, in state-owned and state-holding firms. In private enterprises, with the development of modern corporate governance systems and professional managers, the firm's founders are more willing to let experienced and capable management and decision-making teams operate and manage the company. Therefore, the age of the firm's decision-making and management team tends to be younger, the age gap becomes smaller. According to the distribution results of the chair-CEO age gap, 47.3% of CEOs are post-70 s and 53.3% of the chairman are post-60 s, while post-50 s chairman and CEO only 1.66% and 0.22%, and post-80 s chairman and CEO only 0.13% and 0.26% respectively. These results illustrate that it is statistically significant that 10 years is more appropriate to describe the generational difference between the CEO and chairman of Chinese listed firms. In addition, the squared age gap (i.e., the squared age difference between the CEO and the chairman) and the absolute chair-CEO age gap (i.e., the absolute value of the age difference between the CEO and chairman) are considered. These two variables are used to verify that the age dissimilarity is as important as its sign. In addition, the absolute chair-CEO age gap is used with Chair younger to further test the effect of the sign of age difference. The reason is both the age dissimilarity and its sign will affect the pay gap. Firstly, chair-CEO age gaps are used as independent variables to verify the effect of age dissimilarity on salary incentives, while the absolute chair–CEO age gap and chair younger are used to confirm the impact of the sign of the age dissimilarity on chair-CEO pay gap. In addition, the chair-CEO pay gap is replaced with the CEO-TMT pay gap to test whether chair-CEO age gaps can impact the pay gap between the CEO and other executives. Secondly, the interaction between age difference and CEO power is used to confirm the inhibitory effect of age dissimilarity on the relationship between CEO power and pay gap.

### 2.3 Joint tenure, CEO ability, and chair-CEO age dissimilarity

In China, CEO abilities and relationships are equally important, and they both affect compensation incentives and board supervision. Therefore, we consider that the co-working time between chairman and CEO will reduce the effect of age dissimilarity on the relationship between the CEO power and the chair-CEO pay gap, that is, the co-working time helps CEO to increase mutual communication and reduce conflicts with the chairman. WTD is used as a categorical variable and divides the sample into good relationship group and poor relationship group. The sub-samples of the good relationship group and the poor relationship group accounted for 21.2% and 78.8% of the total sample respectively. It provides evidence that good relationships can reduce the cognitive conflict between the chairman and the CEO, leading to more compensation. In the same way, we also consider that the higher managerial ability of the CEO reduces the effect of age dissimilarity on the relationship between the CEO power and the chair-CEO pay gap. CAD is used as a categorical variable and divides the sample into high-ability CEOs and low-ability CEOs in order to observe the impact of CEO ability on cognitive conflicts caused by age dissimilarity. The sub-samples of high-ability CEOs and low-ability CEOs accounted for 15.8% and 84.2% of the total sample respectively. It provides evidence that CEO with higher ability receives lower constraint from the chairman or board of directors and reward more compensation.

### 2.4 Robustness tests

Heckman two-stage test is used to address the potential endogeneity issue caused by sample selection errors. Reform and opening-up is an important turning point in China's economic development, thus, the instrumental variable Age1, which is a dummy variable set to one if the chairman or the CEO was an adult (16 years old) before 1978, and zero otherwise, is established. It is used to reflect whether senior executives have experienced this special period and the impact of this experience on corporate governance. Harjoto et al. [10] find that the balanced control mechanism will overcome the characteristics difference of the board and reach consensus. Thus, board-CEO age dissimilarity (i.e., age dissimilarity between the CEO and the entire board of directors) as an alternative to chair-CEO age dissimilarity is used to observe whether age gaps have the same effect. Board-CEO age dissimilarity was defined in three ways: board-CEO age gap, board-CEO age ratio, and board-CEO generational gap. The broad age gap is mainly the absolute and relative age difference between the CEO and average age of the board. Board-CEO generational gap is a dummy variable set to one if the age difference between the average of board and CEO is at least 10 years, and zero otherwise. Based on the calculations, the mean age gap between the board and the CEO (Board-CEO age gap) is 2.105 years and the mean age multiple between the board and the CEO (Board-CEO age ratio) is 1.053. The generational gap between the chairman and the CEO(Generational gap) was found in 5.5% of sample firms. From the distribution characteristics of age differences, the board-CEO age dissimilarity also has similar statistical characteristics of the chair-CEO age dissimilarity. And we use the regression equation to further verify the effect of board-CEO age dissimilarity on the relationship between the chair-CEO pay gap and CEO power. Thirdly, the most important endogeneity test consists of treating the 2014 Chinese Salary limitation Order as an exogenous shock and alters the optimal levels of monitoring. Therefore, using 2014(Age2) as a categorical variable, the sample is divided into two sub-samples: Pre-pay curbs (2009–2013) and Post-pay curbs (2014–2018). The sub-samples of Pre-pay curbs and Post-pay curbs accounted for 50.1% and 49.9% of the total sample respectively. Based on the comparison of two sub-samples, we can compare the effect of age dissimilarity on salary incentives before and after the salary limitation order. We use this method mainly to consider the following two aspects: First, although the salary limitation order is only for state-owned and state-controlled listed firms, due to China's special regulatory system and public opinion mechanism, this policy will inevitably affect the entire market. Second, because the existing compensation system and corporate governance are not completely matched, non-state-owned and state-controlled firms also hope to use this opportunity to reform the firm's compensation incentive plan to meet the implementation of national policies.

## Declaration of Competing Interest

The authors declare that they have no known competing financial interests or personal relationships which have, or could be perceived to have, influenced the work reported in this article.

## Appendix Supplementary materials

Image, application 1
